# Root Architecture, Biomass Allocation and Size-Related Allometric Relationships of *Porlieria chilensis* (I.M. Johnst.) in a Mediterranean Ecosystem of Central Chile

**DOI:** 10.3390/plants15213390

**Published:** 2026-11-01

**Authors:** Carolain Badaracco, Sergio Donoso, Karen Peña-Rojas, Claudia Espinoza, Miguel Quintanilla, Rodrigo Gangas, Aileen González

**Affiliations:** Mediterranean Forests Laboratory, Faculty of Forestry and Nature Conservation, University of Chile, Avenida Santa Rosa, La Pintana, Santiago 11315, Chile; carolain.badaracco@uchile.cl (C.B.);

**Keywords:** root-to-shoot ratio, belowground biomass, root collar diameter, potential root exploration area, root length

## Abstract

Mediterranean ecosystems in the central region of Chile face seasonal water limitations and increasing aridity. The belowground structure of their woody species remains poorly known. We characterised root system architecture and biomass allocation in 12 adult trees from a single site of the endemic and Vulnerable *Porlieria chilensis*. These trees had to be removed for the expansion of a tailings dam. Each tree was excavated, and its structural roots (≥0.5 cm) were traced to estimate maximum root length, cumulative root length and potential root exploration area (REA). The aboveground (AB), belowground (BB) and total biomass were determined, and the BB/AB ratio was calculated. The root collar diameter (RCD) showed a positive correlation with the estimated maximum root length (Spearman’s ρ = 0.650), the cumulative root length (ρ = 0.804) and REA (ρ = 0.811), and BB with the cumulative root length (ρ = 0.846) and REA (ρ = 0.797). In contrast, BB/AB decreased with RCD (ρ = −0.713; power-model exponent = −0.080): larger trees had more root biomass in absolute terms but proportionally less relative to aboveground biomass. As roots with a diameter less than 0.5 cm were not completely recovered, BB and BB/AB are conservative estimates. These relationships provide preliminary reference values for non-destructive field assessments, root-zone protection and ex situ planting of this species.

## 1. Introduction

Mediterranean-climate ecosystems are characterized by wet winters and warm, dry summers, a seasonality that imposes pronounced water limitation during part of the year [1]. These ecosystems occur in five regions of the world and exhibit high levels of plant richness and endemism [2], but they are also subject to major anthropogenic and climatic pressures [3]. In central Chile, Mediterranean vegetation develops along a climatic gradient in which precipitation is concentrated mainly during the winter months, whereas the spring–summer period presents a prolonged dry season [4]. During recent decades, increasing aridity has intensified water stress in these ecosystems, and widespread deterioration of Mediterranean forests associated with this process has been documented [5]. Understanding the structural characteristics that allow woody species to persist under this seasonal and increasingly limited water availability is therefore a relevant challenge in the ecology of these systems.

Roots play a central role in water and nutrient acquisition, determining, through their morphological and architectural attributes, how plants explore the soil [6,7,8]. Root traits exhibit high diversity and may be related to resource acquisition processes, plant functioning, and interactions with soil [9]. Root system architecture is also plastic: soil moisture, nutrients, temperature, aeration and mechanical impedance modify root growth and branching [8]; for example, increasing soil compaction shortened the main root, increased lateral root length and reduced root biomass and the root-to-shoot ratio in *Acer velutinum* seedlings [10]. Unlike aboveground organs, whose characterization has historically been more accessible, the study of complete root systems remains limited by the difficulties associated with excavation, measurement, and quantification under natural conditions [11]. This limitation is particularly important in woody species, whose root systems may extend spatially beyond the crown projection and occupy different soil strata [12]. Consequently, root characterization is a necessary complement to the study of aboveground traits when seeking to understand the structural strategy of a plant under seasonal water limitation.

Root architecture and biomass distribution constitute related, although not equivalent, dimensions of belowground plant organization [13,14]. The former describes how the root system is distributed spatially and therefore allows characterization of the depth and extent to which an individual explores the soil [6,15]. The latter quantifies the relative investment of biomass in roots, stems, and leaves and may vary with plant size, environment, and evolutionary history [16]. Contemporary evidence further indicates that root traits are not necessarily organized along a single acquisition–conservation functional axis but rather form a multidimensional space incorporating different morphological, anatomical, and functional attributes [17,18]. In Mediterranean vegetation, for example, variation in fine-root traits reflects multiple functional dimensions rather than exclusively a simple gradient between rapid resource acquisition and resource conservation [18]. Therefore, integrating root architecture with biomass distribution provides complementary perspectives for approaching the structural organization of plants, a perspective that is particularly relevant for woody species in Mediterranean environments subject to water limitation [19,20].

*Porlieria chilensis* (I.M. Johnst.) belongs to the family Zygophyllaceae and is an endemic tree species of Chile, with a geographic distribution extending approximately from 29°27′ S to 34°25′ S latitude [21]. It is part of the Mediterranean vegetation of central Chile and is currently classified as Vulnerable under the national species classification system [21]. Water availability may vary markedly among soil horizons and, consequently, the spatial distribution of roots may influence the soil strata potentially accessible to the plant [22]. In *P. chilensis*, there is experimental evidence of hydraulic functioning associated with the root system. Hydraulic lift is the process by which water passively moves from wetter, deeper soil layers toward drier, shallower layers through the roots [23], and it has been associated with root systems capable of using water located at different depths. Muñoz et al. [24] found evidence of this process in *P. chilensis,* associated with a dimorphic root system capable of taking up water from both shallow and deep soil strata. Complementary recent research shows that *P. chilensis* modifies physiological and metabolic attributes during drought and recovery, including changes in intrinsic water-use efficiency and non-structural carbohydrate reserves [25]. These findings highlight the relevance of the belowground dimension for understanding species functioning under water restriction.

The conservation context of the species reinforces the need for this information. In the central region of Chile, the combination of increasing aridity [5] and anthropogenic disturbance, including land-use change and the expansion of infrastructure such as the tailings dam considered in this study, may increase the risk of decline of local *P. chilensis* populations. Conservation measures such as the protection of root zones around retained trees, the transplanting of individuals affected by development projects, and ex situ or restoration planting require quantitative information on how far, how deep and with how much biomass the root systems of the species extend, which is currently unavailable.

Previous studies of *P. chilensis* have addressed hydraulic lift [24], physiological and metabolic responses to drought [25], and above- and belowground functional traits, including the vertical distribution of roots [26]. However, to our knowledge, no study has quantified the relationships between easily measured aboveground attributes, such as root collar diameter (RCD) and crown diameter, as well as the lateral extent and biomass of the root system of adult trees of this species. In other woody plants, such relationships have been widely used to predict belowground dimensions from aboveground size [14,27], and basal diameter and crown diameter are the most informative predictors in shrub biomass allometries [28]. Equivalent relationships for *P. chilensis* would provide a basis for non-destructive field surveys of populations and for the assessment of habitat and root-zone requirements, which is difficult to obtain otherwise because complete root excavation is destructive and is only justified when trees must be removed.

In this context, the objective of this study was to characterize the distribution of aboveground and belowground biomass and root system architecture in adult *Porlieria chilensis* trees in a Mediterranean ecosystem of central Chile. Specifically, we evaluated the relationships among individual dendrometric attributes, such as aboveground, belowground, and total biomass, and the spatial extent of the root system, and we described the form of the relationships between RCD and estimated maximum root length, potential root exploration area and the belowground-to-aboveground biomass ratio. This approach allows the magnitude of structural investment in roots and its spatial organization to be assessed jointly, providing a quantitative characterization of the belowground dimension of *P. chilensis*.

## 2. Materials and Methods

### 2.1. Study Area

The study was conducted in the Cordillera El Melón, located in the El Soldado mine area, in the municipality of Nogales, Valparaíso Region, Chile, currently owned by Anglo American Chile. The study area is located within the Mediterranean climate zone of central Chile, characterized by marked climatic seasonality, with precipitation concentrated during winter and warm, dry summers. The vegetation corresponds to Mediterranean sclerophyllous forest and shrubland, where *Porlieria chilensis* is one of the representative woody species and occurs mainly on rocky slopes and soils with limited water availability.

### 2.2. Individual Selection and Dendrometric Characterization

Twelve adult *Porlieria chilensis* trees with no apparent damage were studied. All trees were located within the area authorized for the expansion works of the El Torito tailings dam (Exempt Resolution No. 163/2004), where all *P. chilensis* individuals were to be removed as part of these works, and the excavation of their root systems took advantage of this authorized removal. Among the individuals present in this area, the 12 trees were selected to represent the range of tree sizes occurring at the site. The number of trees was limited by the effort required for complete excavation of mature root systems. No selection criteria based on tree vigour or on the presence of neighbouring trees were applied. Microhabitat attributes (e.g., rock fracturing or local soil depth) and the identity, size and distance of neighbouring plants were not recorded.

Before excavation, each tree was characterized by total height (m), measured from the base of the stem to the crown apex, root collar diameter (RCD, cm), and crown radius (m) in the four cardinal directions (north, south, east, and west). From these measurements, north–south and east–west crown diameters were calculated for each tree and subsequently used to analyze their relationships with root architecture and biomass variables.

### 2.3. Excavation and Characterization of Root Architecture

Following dendrometric characterization, trees were excavated using a backhoe to expose and recover the greatest proportion of the root system. Excavation was conducted progressively around each plant, carefully removing soil until the structural roots were exposed. Subsequently, soil adhering to the roots was manually removed to facilitate root identification, measurement, and recording of root-system variables.

Once each tree had been extracted, each structural root was traced from the centre of the plant (stump) toward its distal end. This process was continued until its diameter decreased to approximately 0.5 cm. The 0.5 cm threshold therefore refers to the distal (final) diameter at which the tracing process was ended. Roots with a diameter less than 0.5 cm, which include the fine roots (<2 mm), were not traced. For each structural root, the following variables were recorded: initial diameter (DI, cm), measured at the point of insertion at the stump; final diameter (DF, cm), measured at the distal end of the recovered portion; root length, corresponding to the actual path following its contour; geometric distance, defined as the straight-line distance between the point of insertion at the stump and the distal end of the recovered portion; horizontal distance, obtained as the projection of the geometric distance onto a horizontal plane; depth reached by the root relative to the root collar; and main orientation, measured with a compass from the point of insertion to the most distant point of the root. These procedures follow those described by Peña-Rojas et al. [29] for the excavation of the root systems of adult *Beilschmiedia miersii* trees in central Chile, in which each main root was followed from the stem to a minimum diameter of approximately 0.5 cm and its length along the contour, geometric distance, depth and compass orientation were recorded.

From the set of structural roots of each individual, the root reaching the greatest depth relative to the root collar was identified, and its depth was recorded as maximum depth (MD_max_, m). The final diameter of that root was recorded as the final diameter at maximum depth (FDD_max_, cm); when more than one root reached the same maximum depth, the smallest final diameter among them was recorded.

Because structural roots were traced only to a distal diameter of approximately 0.5 cm and, under the rocky site conditions, frequently broke before reaching this threshold, the distal portion of each root up to its tip could not be recovered. This unrecovered portion was estimated with a projection function relating the projected length of the unrecovered portion up to the root tip (Y, cm) to the final diameter of the recovered portion (x, cm): Y = 122.47x + 27.575 (R^2^ = 0.4403; F_1,9_ = 7.08; *p* = 0.026; n = 11). The function was developed by linear regression from completely recovered roots with final diameters of 0.45–0.55 cm. The projected length was added to the measured length of each of the 182 structural roots to obtain its final estimated length (FEL), and to its measured horizontal distance to obtain its final estimated horizontal distance (FED), assuming that the unrecovered segment continued in the same horizontal direction as the recovered portion. Projected segments accounted for 20.9% of the total estimated root length of the 12 trees. For each tree, the greatest FEL was taken as the estimated maximum root length (L_max_), and the sum of the FEL of all its structural roots as the cumulative root length.

The orientation and FED of each root were used to project the root system onto a horizontal plane and to calculate its potential root exploration area (REA, m^2^), following Peña-Rojas et al. [29]. Each root was represented as a vector from the stump defined by its compass orientation and its FED, and REA was calculated as the area of the polygon formed by joining the distal ends of angularly adjacent roots. REA thus describes the horizontal area potentially occupied by the structural root system, an operational measure of the lateral zone of influence of a plant [27,30]. The number of structural roots of each tree (i.e., the number of roots traced from the stump) was also recorded.

### 2.4. Determination of Aboveground and Belowground Biomass

After root architecture characterization, the 12 individuals were processed for biomass estimation. Aboveground biomass (AB) included leaves, branches and stem. Although foliage and woody material were handled and dried according to their tissue type, the dry mass of the aboveground fraction was recorded as a whole; therefore, AB is reported as a single value per tree, and biomass partitioning among leaves, branches and stem could not be analysed.

Belowground biomass (BB) corresponded to all roots recovered during excavation. Because the study site was characterized by rocky areas, root biomass represented the root system that could be recovered through mechanical excavation, following established approaches for the excavation and characterization of root systems [11,31]. Roots < 0.5 cm in diameter, including fine roots, were not completely recovered: the portions that remained attached to the structural roots were included in BB, but a substantial part was lost during excavation. BB therefore mainly represents the structural (coarse) root system and underestimates total belowground biomass (see Section 4).

All plant material was transported to the laboratory. Leaves and thin branches were placed on trays and dried in a forced-air oven at 70 °C for 48–72 h, cooled in a desiccator, and weighed; they were then subjected to additional 24 h drying cycles, each followed by cooling and weighing, until constant mass was reached. Because the stem, thick branches, stump and thick roots could not be dried whole, their fresh mass was recorded and representative subsamples were taken from each tree. The subsamples were cleaned to remove adhering residues, weighed fresh, and dried following the same procedure until constant mass, and the dry-to-fresh mass ratio of the subsamples was applied to the total fresh mass of the corresponding components to estimate their dry mass [32]. Drying at 70–75 °C rather than at 103–105 °C may leave a small amount of residual water in woody tissues; across eight tree species, dry mass obtained at 75 °C exceeded that obtained at 105 °C by 0.96% for stem wood, 1.76% for branches and 2.20% for foliage [33]. Because the same protocol was applied to all trees and to both biomass fractions, any such bias is expected to be small and to affect AB and BB in the same direction.

Based on these values, total biomass (TB) of each individual was calculated as the sum of aboveground and belowground biomass. The belowground-to-aboveground biomass ratio (Root:Shoot Ratio, R:S) was also determined as the quotient of belowground biomass and aboveground biomass (BB/AB) and was used as an indicator of relative biomass allocation between belowground and aboveground components [11].

### 2.5. Statistical Analysis

All statistical analyses were conducted using the individual tree as the experimental unit (n = 12). Although 182 structural roots were recorded, root-level observations were not treated as independent replicates because multiple roots belonged to the same individual. Root-level measurements were therefore used to derive individual-level descriptors of root architecture, including estimated maximum root length, cumulative root length, and root exploration area.

Descriptive statistics were calculated for dendrometric variables, root architecture attributes, biomass components, and the belowground-to-aboveground biomass ratio (BB/AB). For each variable, the mean, standard deviation, median, minimum and maximum values and coefficient of variation were calculated.

Associations among dendrometric characteristics, root architecture, and biomass variables were evaluated using Spearman’s rank correlation coefficient (ρ); tied values were assigned mid-ranks. This non-parametric approach was selected because of the small sample size and because the analyses focused on monotonic associations among individual-level variables. The main associations evaluated included relationships between RCD, tree height, and crown diameter and root architecture attributes, as well as relationships among dendrometric variables, biomass components, and root exploration attributes.

To further characterize the structural attributes of the longest roots, the four roots with the greatest final estimated length were identified for each individual. For these roots, the mean initial diameter, mean final diameter, mean final estimated length, mean final estimated horizontal distance, and mean depth were calculated for each tree. These individual-level means were subsequently related to dendrometric, root architecture, and biomass variables using Spearman’s rank correlation coefficient, while maintaining the individual tree as the statistical unit.

Additionally, a simple linear regression was fitted to evaluate the relationship between root collar diameter (RCD) and estimated maximum root length. Model adequacy was evaluated using four criteria: (i) linearity of the relationship between RCD and estimated maximum root length, through graphical inspection of the data; (ii) normality of residuals, using the Shapiro–Wilk test; (iii) homogeneity of residual variance, through graphical inspection of residuals versus fitted values; and (iv) the presence of potentially influential observations, through analysis of residuals and Cook’s distance. Once model adequacy had been assessed, the regression coefficient (slope), coefficient of determination (R^2^), F statistic, and associated *p*-value were reported. Statistical significance was assessed at α = 0.05.

To describe the form of the relationships between RCD and the variables REA and BB/AB, two additional regression models were fitted. For potential root exploration area (REA), a second-order polynomial model (REA = β_0_ + β_1_ × RCD + β_2_ × RCD^2^) was fitted to describe a possible non-linear increase in REA with plant size. For the belowground-to-aboveground biomass ratio (BB/AB), a power (allometric) model of the form BB/AB = a × RCD^b^ was fitted by ordinary least squares on log-transformed data (ln BB/AB = ln a + b × ln RCD), an approach widely used to analyse size-dependent changes in biomass distribution [34,35]; in this model, b < 0 indicates a decrease in BB/AB with increasing RCD. Both models were interpreted only within the observed range of RCD (2.3–31.0 cm). Model adequacy was assessed using the same four criteria described above. The coefficients, R^2^, F statistic and *p*-value are reported for each model, and the 95% confidence interval is reported for the exponent of the power model.

Because part of the root length was estimated with the projection function, the main correlations and the linear regression involving root length were repeated using measured root lengths only (i.e., without the projected segments) as a sensitivity analysis.

Analyses, including the sensitivity analysis, were performed using InfoStat version 2020 (Grupo InfoStat, FCA, Universidad Nacional de Córdoba, Córdoba, Argentina) [36].

## 3. Results

### 3.1. Dendrometric Characteristics and Root Architecture

Descriptive statistics of the dendrometric and root architecture variables of the 12 *Porlieria chilensis* trees are presented in Table 1 and the values of each tree in Tables S1 and S2.

The mean height of the trees was 3.19 ± 1.10 m (range: 1.70–5.20 m), whereas mean RCD was 11.35 ± 9.82 cm (range: 2.30–31.00 cm). Crown diameter was 2.78 ± 1.58 m in the north–south direction and 2.42 ± 1.50 m in the east–west direction (Table 1).

Root architecture attributes showed substantial variation among trees. Estimated maximum root length ranged from 3.34 to 13.84 m, whereas cumulative root length ranged from 14.31 to 237.48 m. Potential root exploration area ranged from 3.95 to 153.52 m^2^. The maximum values for estimated maximum root length, cumulative root length, and exploration area occurred in the same individual (tree 3), whereas tree 7 showed the lowest estimated maximum root length, cumulative root length and exploration area. The number of structural roots per tree ranged from 4 to 30, and maximum rooting depth ranged from 0.30 to 1.39 m (Table 1 and Table 2).

RCD was positively associated with estimated maximum root length (ρ = 0.650; *p* = 0.022), cumulative root length (ρ = 0.804; *p* = 0.002) and root exploration area (ρ = 0.811; *p* = 0.001). Crown diameters were also positively associated with estimated maximum root length and, more strongly, with REA (ρ = 0.848 and 0.867 for the north–south and east–west directions, respectively; both *p* < 0.001), whereas tree height was associated with REA (ρ = 0.650; *p* = 0.022) but not with estimated maximum root length (ρ = 0.497; *p* = 0.101) (Table 3; full correlation matrix in Table S3).

**Figure 1:**
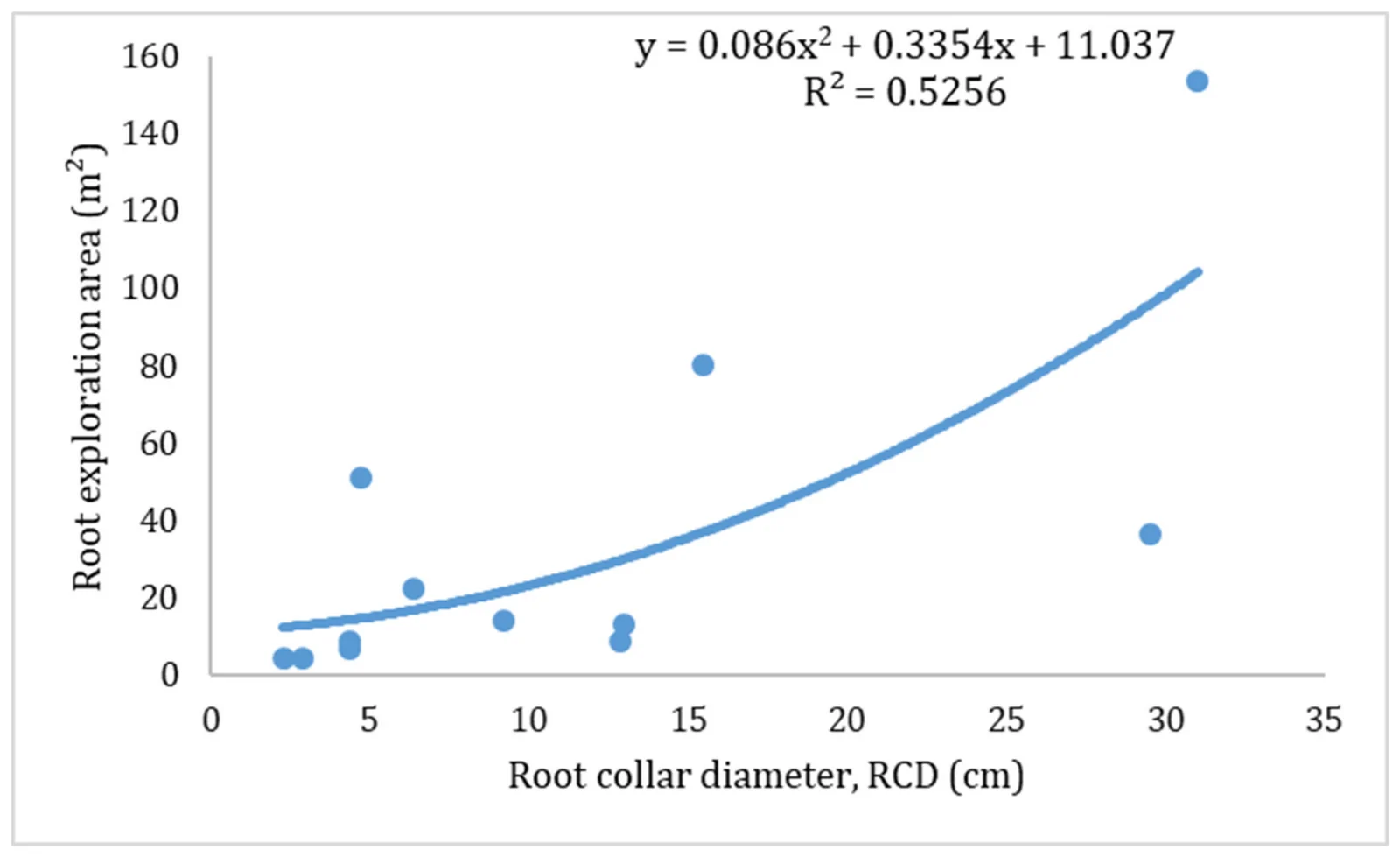
Relationship between root collar diameter (RCD) and potential root exploration area (REA) in 12 adult *Porlieria chilensis* trees from the Cordillera El Melón, central Chile. Each point represents one tree. The line shows the fitted second-order polynomial model REA = 0.086 × RCD^2^ + 0.335 × RCD + 11.037 (R^2^ = 0.5256; *p* = 0.035), interpreted only within the observed range of RCD.

The relationship between RCD and REA was described by a second-order polynomial model (REA = 0.086 × RCD^2^ + 0.335 × RCD + 11.037; R^2^ = 0.5256; adjusted R^2^ = 0.420; F_2,9_ = 4.99; *p* = 0.035), in which REA increased from 12.3 m^2^ to 104.0 m^2^ across the observed range of RCD (Figure 1; Table 4).

However, neither the linear (*p* = 0.94) nor the quadratic term (*p* = 0.53) was individually significant, residual variance increased with plant size, and trees 3 and 12 were identified as influential observations (Cook’s distance = 2.26 and 1.38, respectively). Dispersion was also considerable among trees of similar size; for example, tree 9 (RCD = 4.7 cm) reached 50.79 m^2^, whereas tree 12 (RCD = 29.5 cm) reached 36.39 m^2^. The linear regression between RCD and estimated maximum root length (L_max_) showed a positive relationship (L_max_ = 3.492 + 0.296 × RCD; R^2^ = 0.612; F_1,10_ = 15.76; *p* = 0.0026), with normally distributed residuals (Shapiro–Wilk W = 0.976; *p* = 0.964) and no observation with Cook’s distance > 0.3 (Table 4). Nevertheless, this regression also depended on the largest individuals, and the exclusion of trees 3 and 12 rendered it non-significant (R^2^ = 0.086; *p* = 0.41). Although neither tree was influential according to Cook’s distance, together they defined the upper portion of the sampled size range, so the fitted slope reflects mainly the contrast between these two individuals and the remaining, considerably smaller trees.

When the projected segments were excluded and only measured root lengths were used, the associations were similar (RCD–estimated maximum root length: ρ = 0.636, *p* = 0.026; RCD–cumulative root length: ρ = 0.825, *p* = 0.001; BB–cumulative root length: ρ = 0.811, *p* = 0.001), as was the linear regression between RCD and maximum root length (R^2^ = 0.613; *p* = 0.003).

### 3.2. Biomass Distribution

Aboveground, belowground and total biomass varied widely among trees (CV > 100%), whereas the BB/AB ratio showed much lower relative variation (CV = 11.1%), ranging from 0.320 to 0.431 (Table 5; individual values in Table S1).

RCD was positively associated with aboveground (ρ = 0.755; *p* = 0.005), belowground (ρ = 0.748; *p* = 0.005) and total biomass (ρ = 0.741; *p* = 0.006). Belowground biomass was positively associated with root exploration area (ρ = 0.797; *p* = 0.002) and cumulative root length (ρ = 0.846; *p* < 0.001), whereas RCD was negatively associated with the BB/AB ratio (ρ = −0.713; *p* = 0.009) (Table 3).

**Figure 2:**
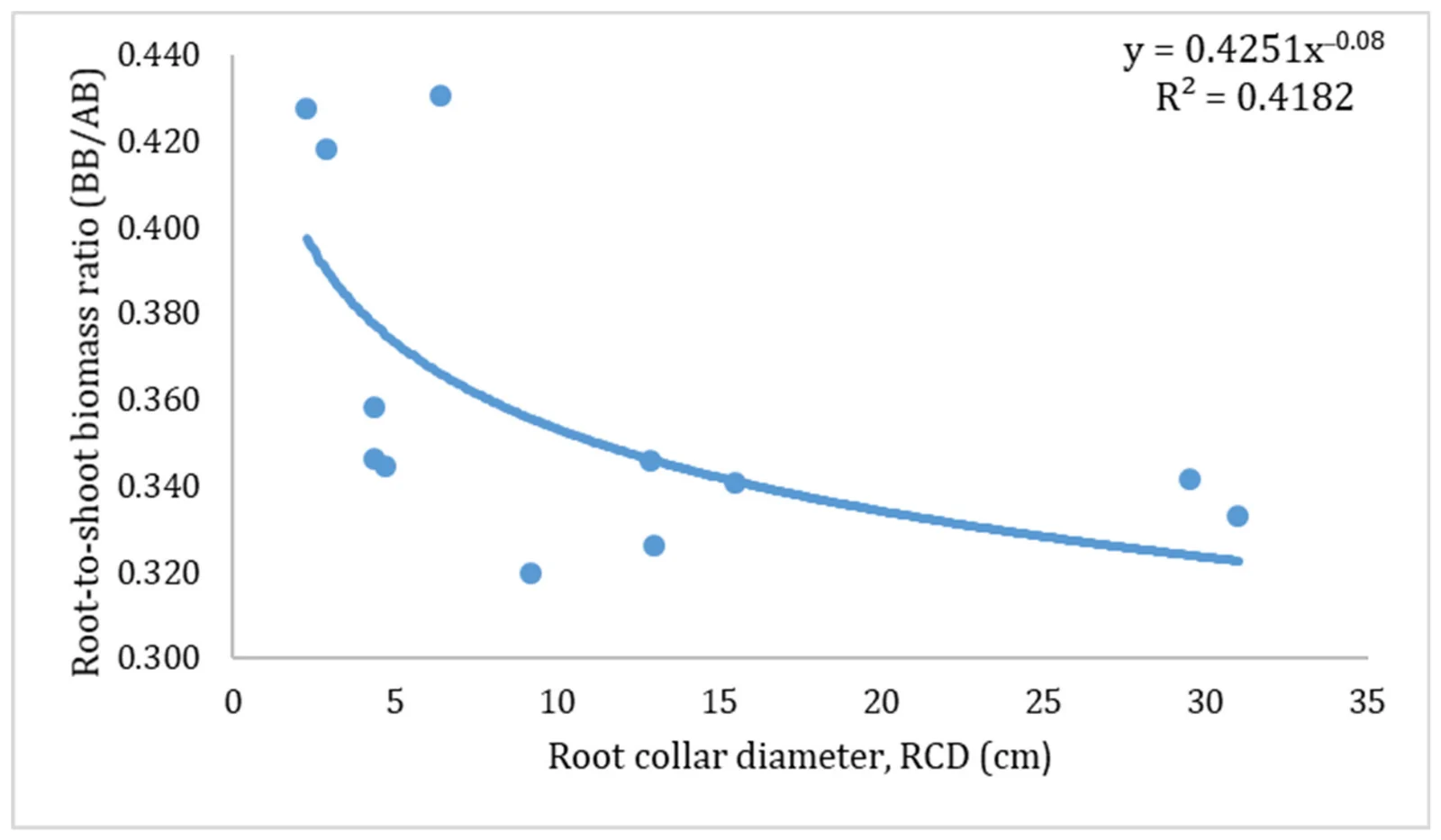
Relationship between root collar diameter (RCD) and the belowground-to-aboveground biomass ratio (BB/AB) in 12 adult *Porlieria chilensis* trees. Each point represents one tree. The line shows the fitted power model BB/AB = 0.425 × RCD^−0.080^; R^2^ = 0.418; *p* = 0.023.

The decrease in BB/AB with RCD was described by a power model with a negative exponent (BB/AB = 0.425·RCD^−0.080^; R^2^ = 0.418; F_1,10_ = 7.19; *p* = 0.023; 95% CI of the exponent: −0.147 to −0.014), with predicted values declining from 0.40 in the smallest tree to 0.32 in the largest (Figure 2; Table 4).

### 3.3. Characteristics of the Longest Roots

For each individual, the four roots with the greatest final estimated length were selected. The mean length of these roots ranged from 2.44 to 13.49 m, whereas mean final estimated horizontal distance and mean depth ranged from 1.88–8.96 m and 0.23–0.91 m, respectively (Table 2).

The mean initial diameter of the four longest roots was positively associated with RCD (ρ = 0.620; *p* = 0.032), height (ρ = 0.623; *p* = 0.030), aboveground biomass (ρ = 0.918; *p* < 0.001), belowground biomass (ρ = 0.900; *p* < 0.001), and total biomass (ρ = 0.911; *p* < 0.001), whereas mean final diameter was positively associated with aboveground biomass (ρ = 0.681; *p* = 0.015), belowground biomass (ρ = 0.726; *p* = 0.007), and total biomass (ρ = 0.719; *p* = 0.008).

The mean length of the four longest roots was positively associated with RCD (ρ = 0.685; *p* = 0.014), north–south crown diameter (ρ = 0.732; *p* = 0.007), east–west crown diameter (ρ = 0.685; *p* = 0.014), root exploration area (ρ = 0.860; *p* < 0.001), and cumulative root length (ρ = 0.783; *p* = 0.003).

Mean final estimated horizontal distance was also positively associated with RCD (ρ = 0.629; *p* = 0.028), north–south crown diameter (ρ = 0.739; *p* = 0.006), east–west crown diameter (ρ = 0.664; *p* = 0.018), and root exploration area (ρ = 0.839; *p* < 0.001).

Mean depth of the four longest roots was positively associated with estimated maximum root length (ρ = 0.692; *p* = 0.013).

## 4. Discussion

The results show a close association between root collar diameter (RCD) in *Porlieria chilensis* and the spatial extent of its root systems. Root collar diameter (RCD) was positively related to estimated maximum root length, cumulative root length, and root exploration area, and the relationship between RCD and estimated maximum root length was also linear and significant. Overall, these results indicate that larger individuals develop structurally more extensive root systems. This pattern is consistent with global evidence from Tumber-Dávila et al. [14], who, based on a dataset of 5647 observations, found strong covariation between the size of aboveground and belowground structures and indicated that shoot size is an important predictor of lateral root system extent. In woody plants, moreover, root system diameter was, on average, approximately twice as large as the width of the aboveground system.

The relationship between RCD and REA (Figure 1) indicates that the potential area explored by roots increased with stem size. The fitted polynomial model described an increase in REA across the observed range of RCD; however, evidence for curvature was limited because the quadratic term was not statistically significant and the model was strongly influenced by the largest individual (tree 3). The considerable dispersion around this relationship further suggests that factors other than tree size contribute to variation in REA among individuals of similar size. For example, tree 9 (RCD = 4.7 cm) explored an area 1.4 times larger than that of tree 12 (RCD = 29.5 cm), and about six times larger than that of tree 10, which had a similar RCD (4.4 cm; REA = 8.45 m^2^). At a global scale, maximum lateral root spread correlates with canopy size, but for a given canopy size it is greater in arid environments and coarse-textured soils [30]. In rocky substrates, roots can grow along fractures and use water stored in weathered bedrock [37], so the spatial heterogeneity of soil depth and rock fracturing could favour long roots in some directions and restrict them in others. Neighbourhood competition is a second plausible source of variation: in a tropical planted forest, more intense competition in the near neighbourhood reduced root system extent in four of five species [31]. Because microhabitat attributes and neighbouring plants were not recorded in the present study, these explanations remain hypotheses. Future studies should combine root excavation or non-destructive root detection with explicit records of rock fracturing, soil depth and neighbourhood composition (e.g., distance-dependent competition indices) to explain the variation in root architecture among individuals of the same size class.

The observed relationship between RCD and root architecture can be interpreted in terms of the potential capacity for spatial soil exploration. Root architecture describes the spatial configuration of the root system and constitutes an important component of plants’ capacity to explore soil and acquire resources under suboptimal environmental conditions [6]. In *P. chilensis*, the increase in root length and potential exploration area with individual size suggests that aboveground growth is accompanied by an expansion of the space occupied belowground. However, this relationship should be interpreted as a modification of the spatial structure of the root system rather than as direct evidence of greater water or nutrient uptake, because these functions were not measured in the present study.

The positive association between belowground biomass, cumulative root length, and root exploration area adds a second dimension to this pattern. Belowground biomass was positively related to both cumulative root length and exploration area, indicating that individuals with greater root biomass also tended to have more spatially extensive systems. Nevertheless, biomass and architecture represent related but non-equivalent attributes of belowground organization. Sinacore et al. [13], through complete tree excavations, demonstrated that species may differ simultaneously in root biomass and architecture and that patterns of horizontal root extension do not necessarily reproduce crown configuration. Consequently, the relationship found in *P. chilensis* suggests coordination between the magnitude of belowground biomass and the space potentially occupied by roots, but it does not establish that one dimension causally determines the other.

The pattern of relative allocation introduces an important distinction from an interpretation based exclusively on absolute biomass. The BB/AB ratio decreased significantly with RCD, although belowground biomass increased with individual size and with cumulative root length and root exploration area. Thus, larger individuals had more root biomass in absolute terms, but this represented a smaller proportion of their total biomass. This result is consistent with the need to distinguish between absolute and relative changes in biomass allocation. The global analysis by Ledo et al. [38], based on 3416 trees from 212 species, showed that the root:shoot ratio is associated with tree size and environmental variables, including climatic water deficit. The negative exponent of the power relationship between RCD and BB/AB (Figure 2) is consistent with the size-dependence of biomass distribution described across c. 1200 species, in which allocation fractions change dynamically as plants grow [34]. More recently, a global synthesis of nearly 30,000 root:shoot observations found that greater plant height reduces R:S, whereas drier conditions, sandy soils and deeper rooting increase it [39]. Notably, the mean BB/AB of *P. chilensis* (0.361) was higher than the median reported for woody plants in that synthesis (≈0.25), which may reflect the water-limited and rocky conditions of the study site; however, differences in root recovery methods among studies limit direct comparison. Likewise, Poorter et al. [16] indicated that biomass distribution among leaves, stems, and roots responds to multiple factors, including plant size and environmental conditions.

Several non-exclusive mechanisms may underlie the decline in BB/AB with size. First, an allometric and ontogenetic component is expected: as woody plants grow, an increasing fraction of biomass accumulates in stems and branches that provide mechanical support and height, so that root:shoot ratios commonly decrease with size even when root biomass continues to increase [34,38]. Second, competition for light favours investment in height and crown expansion as trees grow, and plant height is one of the strongest negative predictors of R:S at the global scale [39]. Third, under seasonal drought, a relatively high early investment in roots may favour access to water during establishment; drought generally increases the root mass fraction [40], and the high BB/AB values of the smallest *P. chilensis* trees are consistent with this possibility, although the present observational data cannot test it. Fourth, and importantly, the ratio may be affected by methodology. Because roots < 0.5 cm, including fine roots, were not completely recovered, BB represents mainly the structural root system. Fine roots can constitute a large share of belowground biomass in water-limited ecosystems; in a global budget, fine roots represented about 74% of root biomass in deserts and about 11% in woodlands and shrublands [41]. If the fine-root fraction differs systematically with tree size, the slope of the BB/AB–RCD relationship could be biased, and in any case the values reported here underestimate total belowground investment. Therefore, the decline in BB/AB should be interpreted as a decline in the relative investment in the structural root system. In this context, the decrease in BB/AB observed in *P. chilensis* should not be interpreted as a reduction in the importance of the root system as individual size increases. Rather, both results, the increase in absolute root biomass and the decrease in its relative proportion, describe different dimensions of the same growth pattern.

The results obtained for the four longest structural roots of each tree (those with the greatest final estimated length) further clarify this relationship at a finer structural scale. Mean length and mean horizontal distance of these roots increased with RCD and crown dimensions, and both variables were also closely related to root exploration area. In contrast, mean depth of the four longest roots showed a significant association only with estimated maximum root length. Overall, this pattern indicates that differences among individuals in the extent of their longest structural roots were strongly associated with the horizontal size of the root system. However, these results do not allow a general lateral growth strategy to be established for *P. chilensis* because the study did not directly compare horizontal and vertical structural investment or evaluate the mechanisms determining root distribution along the soil profile.

Available evidence for *P. chilensis* provides relevant context for interpreting this spatial organization. Morales et al. [26] described dimorphic root systems for this species, characterized by lateral roots extending horizontally and one or more descending roots capable of reaching greater depths. In their study conducted in Bosque Fray Jorge National Park, most of the root biomass of *P. chilensis* was concentrated in the upper 40 cm of soil, while approximately 80% of the roots were distributed within this layer. These findings indicate that the species can combine a substantial presence of roots in shallow soil layers with roots capable of reaching deeper strata. Muñoz et al. [24], in turn, found evidence of hydraulic lift in *P. chilensis*, a process through which water can passively move from wetter, deeper soil layers toward drier surface layers through the root system. However, these studies were conducted at localities and under environmental conditions different from those of the present study. Therefore, although they are compatible with a root organization that allows the use of water located at different depths, they do not permit direct attribution of the patterns observed in this study to mechanisms of differential water uptake.

The relevance of this belowground organization is particularly important in the context of climate change in Mediterranean ecosystems of central Chile. Miranda et al. [5] documented widespread and abrupt deterioration of Mediterranean forests in Chile associated with intense drought and a regional aridification process, while also highlighting uncertainty regarding ecosystem recovery under a deteriorating water balance. In *P. chilensis*, Estay et al. [25] demonstrated that drought induces physiological and metabolic adjustments, including increased intrinsic water-use efficiency, mobilization of starch reserves, and changes in carbohydrate metabolism during stress and recovery. These findings indicate that the response of *P. chilensis* to water restriction may involve both physiological adjustments and changes in the structural organization of the plant. Our results complement this evidence by showing that individual size is closely associated with root system extent and with an increase in absolute belowground biomass. Nevertheless, because the present study is observational, these patterns cannot be interpreted as responses directly induced by climate change or drought.

Interpretation of these results should consider several methodological limitations. First, because roots were traced only to a distal diameter of ≈0.5 cm and frequently broke under the rocky site conditions, their distal portions up to the root tip were estimated using a projection function fitted to a small number of roots (n = 11) and with a moderate coefficient of determination (R^2^ = 0.4403), which accounted for about one-fifth of the total estimated root length. This uncertainty mainly affects cumulative root length and potential exploration area, although the main associations were similar when only measured root lengths were used. Second, roots < 0.5 cm, including fine roots, were not completely recovered, so BB and BB/AB underestimate total belowground biomass and investment. Third, AB was recorded as a single value, which prevented the analysis of biomass partitioning among leaves, branches and stem, and the 70 °C drying temperature may have left a small amount of residual water in woody tissues. Fourth, the sample size was small (n = 12) and all trees came from a single area, which limits the ability to generalize the observed associations to other populations of *P. chilensis*. Moreover, trees were selected to cover the range of sizes present rather than at random, which is appropriate for describing size-related relationships but means that the sample is not necessarily representative of the size distribution of the local population. With small samples, individual observations may substantially influence the magnitude of the coefficients, as shown by the sensitivity of the RCD-L_max_ regression to the two largest trees (Section 3.1). Therefore, the relationships identified should be regarded as patterns of association within the set of individuals studied rather than as universal relationships for the species, and the fitted models (Figure 1 and Figure 2; Table 4) should be interpreted cautiously and only within the observed range of RCD. Finally, microhabitat attributes and neighbourhood competition were not recorded, so their contribution to the variation among trees could not be evaluated. A methodological strength of the study is that the individual tree was retained as the experimental unit, avoiding treatment of the 182 structural roots recovered as independent replicates. In addition, the study took advantage of an exceptional opportunity, the authorized removal of *P. chilensis* trees from the footprint of the tailings dam expansion, to excavate complete structural root systems of a threatened species, which would otherwise not be ethically or legally justifiable.

Overall, the results show that the belowground organization of *P. chilensis* is closely associated with individual size. Individuals with greater RCD have more extensive root systems and greater absolute belowground biomass, while the relative proportion of biomass allocated to the belowground structural component decreases simultaneously. The association between belowground biomass, cumulative root length, and root exploration area further indicates that the magnitude of belowground structure and the space potentially explored covary within the individuals studied. From an applied perspective, the relationships between RCD or crown diameter and root extent provide the first reference values for non-destructive field assessments, such as defining minimum root-protection zones around retained trees, planning the size of root balls for the transplanting of individuals affected by development projects, and estimating belowground biomass in conservation and restoration plantings. These applications should be validated with larger samples from other populations before being used as predictive tools. In the context of increasing aridification of Mediterranean ecosystems in central Chile, these results provide a quantitative characterization of the belowground dimension of *P. chilensis* and a basis for incorporating root architecture and biomass into future studies aimed at understanding its functioning and persistence under water restriction.

## 5. Conclusions

Root system architecture in *Porlieria chilensis* was positively associated with individual size, as root collar diameter was related to estimated maximum root length, cumulative root length, and potential root exploration area. Belowground biomass was positively associated with cumulative root length and potential root exploration area, indicating a correspondence between root biomass and the spatial extent of the root system. In contrast, the belowground-to-aboveground biomass ratio decreased with increasing root collar diameter, indicating that larger individuals had greater absolute belowground biomass, but belowground biomass represented a smaller proportion relative to aboveground biomass. Because fine roots were not completely recovered, these values describe mainly the structural root system and should be considered conservative estimates of belowground investment.

These results provide baseline parameters for the conservation of *P. chilensis* populations and for restoration and dryland planting of the species in the Mediterranean climate zone of Chile, particularly for estimating root extent from easily measured aboveground traits. Future studies should validate these relationships with larger samples, include the fine-root fraction, and compare root architecture among populations growing in different habitats, recording microhabitat conditions and neighbourhood competition, in order to clarify the mechanisms by which environmental filtering regulates belowground traits in this species.

## Figures and Tables

**Table 1:** Descriptive statistics of dendrometric and root architecture variables of 12 adult *Porlieria chilensis* trees in a Mediterranean ecosystem of central Chile (n = 12).

Variable	Mean ± SD	Median	Minimum	Maximum	CV (%)
Height (m)	3.19 ± 1.10	3.20	1.70	5.20	34.5
Root collar diameter, RCD (cm)	11.35 ± 9.82	7.80	2.30	31.00	86.6
Crown diameter N–S (m)	2.78 ± 1.58	2.20	1.00	6.30	56.8
Crown diameter E–W (m)	2.42 ± 1.50	2.20	0.60	5.30	61.9
Number of structural roots	15.2 ± 8.0	14.5	4.0	30.0	52.7
Estimated maximum root length, L_max_ (m)	6.85 ± 3.72	5.62	3.34	13.84	54.3
Cumulative root length (m)	72.06 ± 65.18	51.81	14.31	237.48	90.5
Potential root exploration area, REA (m^2^)	33.52 ± 44.22	13.51	3.95	153.52	131.9
Maximum rooting depth, MD_max_ (m)	0.77 ± 0.36	0.78	0.30	1.39	46.2

Note: SD, standard deviation; CV, coefficient of variation; RCD, root collar diameter; N–S, north–south; E–W, east–west; L_max_, estimated maximum root length (longest final estimated root length of each tree); REA, potential root exploration area; MD_max_, greatest depth reached by any structural root of each tree. Root length variables include the projected distal segments (Section 2.3).

**Table 2:** Characteristics of the structural roots of each *Porlieria chilensis* tree: four longest roots, deepest root and potential root exploration area.

Tree	FEL (m)	FED (m)	MD (m)	MD_max_ (m)	FDD_max_ (cm)	REA (m^2^)
1	3.49 ± 0.11	2.64 ± 0.40	0.39 ± 0.14	0.59	1.06	8.66
2	4.34 ± 0.76	3.12 ± 0.63	0.60 ± 0.24	0.95	0.47	14.00
3	13.49 ± 0.33	7.88 ± 0.38	0.60 ± 0.12	0.80	0.40	153.52
4	4.47 ± 0.43	2.93 ± 0.09	0.79 ± 0.39	1.10	0.40	13.01
5	5.05 ± 0.83	4.28 ± 0.88	0.39 ± 0.13	0.45	0.40	22.00
6	3.19 ± 0.17	2.67 ± 0.31	0.32 ± 0.10	0.40	0.30	6.74
7	2.44 ± 0.63	1.88 ± 0.41	0.23 ± 0.09	0.30	0.20	3.95
8	4.65 ± 1.69	3.15 ± 0.99	0.66 ± 0.18	0.80	0.50	4.41
9	8.56 ± 0.86	7.63 ± 1.28	0.91 ± 0.39	1.30	0.60	50.79
10	3.18 ± 0.49	2.42 ± 0.45	0.40 ± 0.04	0.45	0.50	8.45
11	9.64 ± 0.73	7.60 ± 0.83	0.45 ± 0.15	0.75	0.40	80.26
12	10.68 ± 1.85	8.96 ± 1.69	0.84 ± 0.39	1.39	1.80	36.39

Note: FEL = final estimated length; FED = final estimated horizontal distance; MD = mean depth; MD_max_ = maximum depth; FDD_max_ = final diameter at maximum depth; REA = potential root exploration area. Values of FEL, FED and MD correspond to the mean ± standard deviation of the four roots with the greatest final estimated length for each individual. MD_max_ and FDD_max_ correspond, respectively, to the greatest depth reached among all recovered roots of each individual and the final diameter of the root that reached that depth; when several roots reached the same maximum depth, the smallest final diameter is given. FEL and FED include the projected distal segment (see Section 2.3).

**Table 3:** Spearman’s rank correlation coefficients (ρ) between dendrometric, root architecture and biomass variables in 12 adult *Porlieria chilensis* trees.

Predictor	Response	ρ	*p*
Root collar diameter (RCD)	Estimated maximum root length	0.650	0.022
	Cumulative root length	0.804	0.002
	Potential root exploration area	0.811	0.001
	Aboveground biomass	0.755	0.005
	Belowground biomass	0.748	0.005
	Total biomass	0.741	0.006
	BB/AB ratio	−0.713	0.009
Crown diameter N–S	Estimated maximum root length	0.722	0.008
	Potential root exploration area	0.848	<0.001
	Belowground biomass	0.792	0.002
Crown diameter E–W	Estimated maximum root length	0.706	0.010
	Potential root exploration area	0.867	<0.001
	Belowground biomass	0.699	0.011
Height	Estimated maximum root length	0.497	0.101
	Potential root exploration area	0.650	0.022
Belowground biomass (BB)	Cumulative root length	0.846	<0.001
	Potential root exploration area	0.797	0.002
BB/AB ratio	Potential root exploration area	−0.559	0.059
	Estimated maximum root length	−0.350	0.265

BB/AB, belowground-to-aboveground biomass ratio; N–S, north–south; E–W, east–west. Tied values were assigned mid-ranks; n = 12 in all cases.

**Table 4:** Regression models describing the relationships between root collar diameter (RCD, cm) and root architecture and biomass allocation variables in 12 adult *Porlieria chilensis* trees.

Response	Model	Fitted Equation	R^2^ (adj. R^2^)	F (df)	*p*	Shapiro–Wilk *p*	Max. Cook’s D (Tree)
L_max_ (m)	Linear	L_max_ = 3.492 + 0.296 × RCD	0.612 (0.573)	15.76 (1, 10)	0.003	0.964	0.29 (9)
REA (m^2^)	Second-order polynomial	REA = 11.037 + 0.335 × RCD + 0.086 × RCD^2^	0.526 (0.420)	4.99 (2, 9)	0.035	0.164	2.26 (3)
BB/AB	Power (log–log OLS)	BB/AB = 0.425 × RCD^−0.080^	0.418 (0.360)	7.19 (1, 10)	0.023	0.569	0.20 (5, 8)

Note: L_max_, estimated maximum root length; REA, potential root exploration area; BB/AB, belowground-to-aboveground biomass ratio; adj., adjusted; df, degrees of freedom; OLS, ordinary least squares. For the power model, R^2^ refers to the log-transformed scale and the 95% confidence interval of the exponent is −0.147 to −0.014. Models are valid only within the observed range of RCD (2.3–31.0 cm).

**Table 5:** Aboveground, belowground and total dry biomass and belowground-to-aboveground biomass ratio of 12 adult *Porlieria chilensis* trees.

Variable	Mean ± SD	Median	Minimum	Maximum	CV (%)
Aboveground biomass, AB (kg)	13.37 ± 13.92	9.51	0.39	45.19	104.1
Belowground biomass, BB (kg)	4.55 ± 4.71	3.15	0.16	15.40	103.5
Total biomass, TB (kg)	17.93 ± 18.63	12.66	0.55	60.59	103.9
BB/AB ratio	0.361 ± 0.040	0.345	0.320	0.431	11.1

Note: SD, standard deviation; CV, coefficient of variation; AB, aboveground biomass (leaves, branches and stem); BB, belowground biomass (recovered roots); TB, total biomass (AB + BB); BB/AB, belowground-to-aboveground biomass ratio.

## Data Availability

The data supporting the findings of this study are included in the article and its Supplementary Materials.

## Supplementary Materials

The following supporting information can be downloaded at: https://www.mdpi.com/article/10.3390/plants15213390/s1, Table S1: Dendrometric, root architecture and biomass data of each *Porlieria chilensis* tree; Table S2: Crown radius of each tree in the four cardinal directions; Table S3: Spearman correlation matrix (ρ) among dendrometric, root architecture and biomass variables of 12 *Porlieria chilensis* trees.
