# Supplementary material for: Root Architecture, Biomass Allocation and Size-Related Allometric Relationships of Porlieria chilensis (I.M. Johnst.) in a Mediterranean Ecosystem of Central Chile
Source: Plants (Basel). 2026 Nov 1;15(21):3390. doi: 10.3390/plants15213390 (PMC13765851; doi:10.3390/plants15213390)
Supplement: Supplementary file 1 [file plants-15-03390-s001.zip › Badaracco et al 2026_Supplementary tables.pdf]

Supplementary Tables

Table S1. Dendrometric, root architecture and biomass data of each *Porlieria chilensis* tree.

Tree	RCD (cm)	H (m)	CD_{NS} (m)	CD_{EW} (m)	N_{roots}	L_{max} (m)	L_{cum} (m)	REA (m^2)	AB (g)	BB (g)	TB (g)	BB/AB
1	12.9	3.5	1.90	1.30	20	3.60	52.22	8.66	20,785.33	7,193.11	27,978.44	0.346
2	9.2	4.6	3.40	3.50	21	5.11	58.77	14.00	21,213.30	6,785.64	27,998.94	0.320
3	31.0	3.7	4.30	5.30	30	13.84	237.48	153.52	13,632.36	4,541.72	18,174.08	0.333
4	13.0	3.1	2.40	2.80	15	4.74	51.39	13.01	5,391.56	1,757.91	7,149.47	0.326
5	6.4	3.3	2.00	1.60	11	6.22	41.67	22.00	2,416.34	1,040.94	3,457.28	0.431
6	4.37	2.8	1.70	0.90	8	3.38	22.77	6.74	910.19	315.13	1,225.32	0.346
7	2.9	1.9	1.90	1.30	7	3.34	14.31	3.95	385.26	161.14	546.40	0.418
8	2.3	1.7	1.00	0.60	4	6.14	18.60	4.41	871.43	372.78	1,244.21	0.428
9	4.7	2.0	2.50	3.00	25	9.46	131.63	50.79	21,760.99	7,498.68	29,259.67	0.345
10	4.4	2.4	1.30	1.10	8	3.61	20.76	8.45	2,389.77	856.44	3,246.21	0.358
11	15.5	4.1	4.60	4.20	19	10.51	115.47	80.26	45,187.44	15,399.84	60,587.28	0.341
12	29.5	5.2	6.30	3.40	14	12.23	99.61	36.39	25,533.95	8,722.71	34,256.66	0.342

Note: RCD, root collar diameter; H, height; CD_{NS} and CD_{EW}, north–south and east–west crown diameter; N_{roots}, number of structural roots; L_{max}, estimated maximum root length; L_{cum}, cumulative root length; REA, potential root exploration area; AB, BB and TB, aboveground, belowground and total dry biomass; BB/AB, belowground-to-aboveground biomass ratio.

Table S2. Crown radius (m) of each *Porlieria chilensis* tree in the four cardinal directions.

Tree	North	South	East	West
1	1.0	0.9	0.7	0.6
2	0.9	2.5	1.1	2.4
3	1.8	2.5	2.5	2.8
4	1.3	1.1	1.1	1.7
5	1.2	0.8	0.7	0.9
6	0.8	0.9	0.5	0.4
7	1.2	0.7	0.8	0.5
8	0.7	0.3	0.2	0.4
9	1.2	1.3	1.0	2.0
10	1.0	0.3	0.9	0.2
11	1.8	2.8	0.7	3.5
12	2.4	3.9	1.3	2.1

Note: Crown radius values are given to one decimal place.

Table S3. Spearman correlation matrix (ρ) among dendrometric, root architecture and biomass variables of 12 *Porlieria chilensis* trees.

	H	CD_{NS}	CD_{EW}	N_{roots}	L_{max}	L_{cum}	REA	MD_{max}	AB	BB	TB	BB/AB
RCD	0.85**	0.84**	0.81**	0.72**	0.65*	0.80**	0.81**	0.52	0.76**	0.75**	0.74**	−0.71**
H		0.79**	0.71**	0.57	0.50	0.65*	0.65*	0.38	0.75**	0.70*	0.72**	−0.64*
CD_{NS}			0.94**	0.69*	0.72**	0.83**	0.85**	0.60*	0.84**	0.79**	0.82**	−0.70*
CD_{EW}				0.77**	0.71*	0.84**	0.87**	0.51	0.75**	0.70*	0.74**	−0.74**
N_{roots}					0.52	0.91**	0.80**	0.53	0.75**	0.72**	0.73**	−0.74**
L_{max}						0.76**	0.87**	0.65*	0.65*	0.66*	0.69*	−0.35
L_{cum}							0.94**	0.64*	0.87**	0.85**	0.85**	−0.70*
REA								0.55	0.82**	0.80**	0.81**	−0.56
MD_{max}									0.66*	0.67*	0.70*	−0.62*
AB										0.99**	0.99**	−0.66*
BB											0.99**	−0.59*
TB												−0.64*

Note: * $p < 0.05$; ** $p < 0.01$. Abbreviations as in Table S1; MD_{max}, maximum rooting depth. Tied values were assigned mid-ranks.
